# Antibody Responses to Antigenic Targets of Recent Exposure Are Associated With Low-Density Parasitemia in Controlled Human *Plasmodium falciparum* Infections

**DOI:** 10.3389/fmicb.2018.03300

**Published:** 2019-01-16

**Authors:** Lotus L. van den Hoogen, Jona Walk, Tate Oulton, Isaie J. Reuling, Linda Reiling, James G. Beeson, Ross L. Coppel, Susheel K. Singh, Simon J. Draper, Teun Bousema, Chris Drakeley, Robert Sauerwein, Kevin K. A. Tetteh

**Affiliations:** ^1^Department of Immunology and Infection, London School of Hygiene & Tropical Medicine, London, United Kingdom; ^2^Department of Medical Microbiology, Radboud University Medical Center, Nijmegen, Netherlands; ^3^Burnet Institute, Melbourne, VIC, Australia; ^4^Department of Medicine, The University of Melbourne, Melbourne, VIC, Australia; ^5^Department of Microbiology, Monash University, Clayton, VIC, Australia; ^6^Department of Congenital Disorders, Statens Serum Institut, Copenhagen, Denmark; ^7^Department of International Health, Immunology and Microbiology, Centre for Medical Parasitology, University of Copenhagen, Copenhagen, Denmark; ^8^Jenner Institute, University of Oxford, Oxford, United Kingdom

**Keywords:** malaria, antibodies, exposure, controlled human malaria infection (CHMI), sero-surveillance, sero-epidemiology

## Abstract

The majority of malaria infections in low transmission settings remain undetectable by conventional diagnostics. A powerful model to identify antibody responses that allow accurate detection of recent exposure to low-density infections is controlled human malaria infection (CHMI) studies in which healthy volunteers are infected with the *Plasmodium* parasite. We aimed to evaluate antibody responses in malaria-naïve volunteers exposed to a single CHMI using a custom-made protein microarray. All participants developed a blood-stage infection with peak parasite densities up to 100 parasites/μl in the majority of participants (50/54), while the remaining four participants had peak densities between 100 and 200 parasites/μl. There was a strong correlation between parasite density and antibody responses associated with the most reactive blood-stage targets 1 month after CHMI (Etramp 5, GLURP-R2, MSP4 and MSP1-19; Spearman’s ρ = 0.82, *p* < 0.001). Most volunteers developed antibodies against a potential marker of recent exposure: Etramp 5 (37/45, 82%). Our findings justify validation in endemic populations to define a minimum set of antigens needed to detect exposure to natural low-density infections.

## Introduction

The use of serological tests to measure antibodies against malaria has been advocated as an adjunct approach to improve the detection of transmission dynamics (Corran et al., 2007; Stewart et al., 2009; malERA Refresh Consultative Panel on Characterising the Reservoir and Measuring Transmission, 2017). This is particularly useful in low transmission settings, where the detection of low-density infections is a major challenge (Okell et al., 2009; Wu et al., 2015). Due to the longevity of antibody responses they cannot be used as a diagnostic for current infections, but at the population-level, when combined with age, they represent historical and recent transmission (Drakeley et al., 2005; Corran et al., 2007; Stewart et al., 2009). Antibody metrics are less influenced by fluctuations in infection rates between seasons, and where infection rates fall to near elimination, they can help determine whether there is any remaining ongoing transmission. The discovery of antigenic markers that correlate with recent microscopic infection shows promise in the context of detecting recent malaria transmission patterns more sensitively (i.e., up to 1 year) (Helb et al., 2015). However, it remains largely unknown which antigens most reliably induce measurable antibody responses to allow accurate detection of recent exposure to low-density infections.

The identification of antibody responses, and their corresponding antigen targets, following low-density infections in endemic settings is challenging, as the history of previous exposure is often difficult to determine. Longitudinal studies have demonstrated the acquisition of antibodies following asymptomatic infection in endemic areas and suggest that antibodies to some antigens might be more sensitive markers of recent exposure (McCallum et al., 2017). A powerful model to examine this is using controlled human malaria infections (CHMI) in which healthy volunteers are infected via mosquito bites (Roestenberg et al., 2009), parenteral injection with sporozoites (Bastiaens et al., 2016) or infected red blood cells (Pombo et al., 2002). Parasite densities are monitored intensely and remain low as treatment is provided either at the first microscopy-detectable parasitemia, or even earlier at levels detectable only by qPCR (Walk et al., 2016). CHMI studies in non-endemic (reviewed in (Sauerwein et al., 2011)) and endemic (Shekalaghe et al., 2014; Hodgson et al., 2015) settings generally aim to determine correlates of immune protection or test vaccination strategies (Bastiaens et al., 2016). Therefore, responses against mainly pre-erythrocytic antigens have been studied (Felgner et al., 2013; Nahrendorf et al., 2014; Peng et al., 2016), some of which have been suggested as markers of recent parasite exposure (Nahrendorf et al., 2014). However, few have studied antibody responses in previously naïve control groups and only a small number of antigenic targets have been analyzed using enzyme-linked immunosorbent (ELISA) or multiplex bead assays (Turner et al., 2011; Obiero et al., 2015; Hodgson et al., 2016; Burel et al., 2017).

Protein microarrays enable the simultaneous detection of antibody responses to hundreds of antigens to identify biomarkers related to protection or exposure (Boyle et al., 2017). Antigen production for these arrays have mostly used the *in vitro* translation/transcription (IVTT) open reading frame (ORF) method – a polymerase chain reaction (PCR)-based approach that generates large numbers of putative proteins (Davies et al., 2005). In this study, we use a custom-made protein microarray based on purified recombinant malaria antigens which was enriched for antigens associated with recent exposure. Using this array, we aimed to identify immunogenic targets associated with recent low-density *Plasmodium falciparum* infections in previously malaria-naïve CHMI participants.

## Materials and Methods

### Study Population

Fifty-four malaria naïve participants [based on patient history and lack of antibody responses to asexual parasite lysate (Walk et al., 2017)] from eight CHMI studies were included (Supplementary Table S1). The study population and sampling frame have been described in detail elsewhere (Bijker et al., 2013, 2014a,b; Bastiaens et al., 2016; Walk et al., 2017; Reuling et al., 2018). In short, volunteers were infected by exposure to five laboratory reared *Anopheles* mosquitoes infected with *P. falciparum* sporozoites of the well characterized NF54 strain, its clone 3D7, or the more recently characterized NF135.C10 (Teirlinck et al., 2013) or NF166.C8 (McCall et al., 2017) strains. Citrate plasma samples for antibody detection were selected at three time points: 1 day pre-challenge (C_-1_), 21 or 35 days after challenge (median 30 days; C_+30_) and 64, 140, or 213 days after challenge (median 115 days; C_+115_). All available samples were analyzed.

### Ethics Statement

All clinical trials were carried out in accordance with Good Clinical Practice guidelines and were prospectively registered at ClinicalTrials.gov (NCT numbers listed in Supplementary Table S1). All subjects gave written informed consent prior to participation in accordance with the Declaration of Helsinki. Each clinical trial protocol was approved by the Central Committee on Research Involving Human Subjects (CCMO) of the Netherlands (reference numbers listed in Supplementary Table S1). Study 8 was also approved by the Western Institutional Review Board (WIRB) in the United States.

### Parasite Detection

Volunteers were monitored for the development of symptoms and blood-stage parasites once or twice daily after infection. In studies 1–4 and 8 (Supplementary Table S1) parasitemia was treated when detectable by thick blood smear. Blood smears were read according to a standardized protocol for CHMI studies (Bijker et al., 2014a), in which slides are scored as positive if at least two parasites were seen in 0.5 μl of blood (threshold of ∼4 parasites/μl). A second independent microscopist confirmed positivity. In these studies qPCR was performed on all blood samples according to a previously published protocol (Schats et al., 2015). In studies 5–7, qPCR was performed prospectively, and volunteers were treated when parasitemia reached the predetermined threshold of 0.1 parasites/μl (Walk et al., 2016). In study 8, some volunteers had a recrudescent infection after initial subcurative treatment (Reuling et al., 2018).

### Protein Microarray

The IgG responses to 40 antigenic targets, all blood-stage related except for one (CSP), were determined using a custom-made protein microarray (see Supplementary Table S2 for antigen details). Protein preparations at a concentration of 100 μg/μl of protein in printing buffer (ArrayJet, Scotland) were spotted onto nitrocellulose coated slides (Grace Bio-Labs, United States) with a glycerol-based buffer using the ArrayJet Marathon printer (ArrayJet, Scotland) at the London School of Hygiene and Tropical Medicine (LSHTM). Each slide was sub-divided into 16 arrays with each array consisting of the full complement of antigenic targets printed in duplicate. A standard curve of total human IgG was printed in duplicate within each array (starting concentration 200 μg/ml, fivefold series of 6 points). Samples were processed for IgG detection at the Radboud University Medical Center in Nijmegen. Serum samples were diluted in a deep well at 1:200 in blocking buffer [Phosphate buffered saline (PBS)/BlockIt buffer (ArrayIt Corporation, United States) 25%]. The printed nitrocellulose slides were placed in multi-well hybridization cassettes (HC; ArrayIt Corporation, United States), blocked with 200 μl of blocking buffer and incubated on a rotary shaker (100 rpm) at room temperature (RT) for 1 h. Slides were washed three times: liquid was removed by sharply flicking buffer into a sink, then 200 μl of wash buffer (PBS/Tween 0.05%) was immediately added and the HC placed on the rotary shaker for 2 min. After the final wash, wash buffer was aspirated using a multichannel pipette one column at the time and 100 μl of test samples was added immediately to avoid drying of the nitrocellulose slides. Participant samples, two positive control pools of hyper-immune sera (three repeats of a Ugandan and four repeats of a Tanzanian pool) and one blank (i.e., blocking buffer only) were distributed over twelve slides. Time points from the same participants were grouped on slides where possible to avoid influences of inter-slide variability during assay processing. Slides were incubated for 1 h at RT on the rotary platform. Slides were washed again three times and IgG-specific goat anti-human secondary antibody (Alexa Fluor 633 goat anti-human IgG; Invitrogen) was added in the same manner as the samples at a concentration of 1:1000. Slides were incubated for 1 h at RT on the rotary platform. After a further three washes, slides were dried by centrifuging them at 3000 rpm for 5 min at RT. Slides were stored at +4°C and read 3 days after assay processing at LSHTM using the GenePix 4300A scanner (Molecular Devices, United States) at a wavelength of 635 nm.

Median fluorescence intensity (MFI) was background-corrected (i.e., local reactivity around the spot; bkg) and duplicate measurements were averaged (Pearson’s correlation coefficient 0.99, *p* < 0.001; Supplementary Figure S1). MFI-bkg values smaller than or equal to zero, were replaced with the average value of blank responses and log-transformed. Printing variability was minimal, as determined by the coefficient of variation (CV) of the third point of the standard IgG curve. Inter-slide variability was measured at 1.4% CV, while intra-slide variability was measured at <2.5%. Likewise, assay variability was minimal as shown by the CV of repeated MFI-bkg values of the positive control pools on different slides for GLURP-R2, MSP4 and CSP (associated with high, medium and low antibody responses): 1.5, 0.2, and 5.5% for the Tanzanian pool (*n* = 4), and 0.5, 0.3, and 4.2% for the Ugandan pool (*n* = 3).

### Statistical Analyses

All statistical analyses were performed in STATA 14 and PRISM 7. Cumulative parasite density was expressed as the log-transformed area under the curve (AUC) for parasite density versus time in days using the *pkexamine* command in STATA with the *trapezoid* option. Only parasite density results up to, and including, the day of curative antimalarial treatment were included. Tertiles were used to categorize low, medium and high cumulative parasite density. Antibody responses (IgG) were expressed as log-transformed MFI-bkg values. The average response of forty-five participants at C_-1_ plus two standard deviations was used as the threshold for seropositivity by antigen. Antibody responses at C_+30_ and C_+115_ were standardized by subtracting the mean and dividing by the standard deviation (SD) of C_-1_ responses. For both, one outlier at baseline for Etramp 4 Ag 2 was removed (log-transformed MFI-bkg over 8). The Cochran-Armitage test was used to test the trend in the proportion of antigenic targets recognized at each time point over categories of cumulative parasite density. Spearman’s rank coefficients (ρ) were used to assess the correlation between antibody responses and cumulative parasite density. The level of statistical significance for individual antigens was adjusted according to the Bonferroni correction. Linear regression was used to test the association between participant characteristics and cumulative parasite density.

## Results

### Cumulative Parasite Density and Peak Parasite Density

**FIGURE 1:**
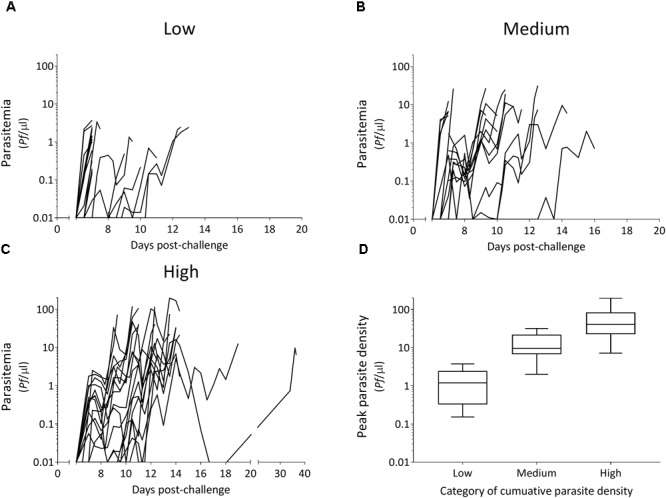
Parasitemia after controlled human malaria infection over categories of cumulative parasite density **(A–C)** and associated peak parasite densities **(D)**. Tertiles of cumulative parasite density (expressed as the log-transformed area under the curve for parasite density versus time until, and including the day of treatment) were used to categorize low **(A)**, medium **(B)**, and high **(C)**. Peak parasite density is the maximum number of parasites/μl detected during the infection until, and including the day of treatment.

All 54 CHMI participants developed a blood-stage infection after sporozoite-induced challenge through infective mosquito bites [median day of first blood-stage parasites detected by qPCR: 7.0, interquartile range (IQR) 6.5–7.0]. Parasitemias ranged from peak parasite densities below 1 parasites/μl that were treated 7 days post-challenge, to peak densities of 198 parasites/μl that were treated 14 days post-challenge, as well as recrudescent infections that lasted 39 days post-challenge (Figures 1A–C). The majority of individuals had peak parasite densities under 100 parasites/μl (50/54), with 19% under one parasite/μl (10/54). A statistically significant difference in cumulative parasite density (expressed as the log-transformed area under the curve for parasite density versus time in days) was seen by gender (*p* = 0.011), which disappeared after adjusting for study (*p* = 0.861). The median age was 21 (IQR 19–22) and did not differ between categories of parasite exposure (*p* = 0.541). As expected, a statistically significant increase was seen in peak parasite density with increasing cumulative parasite density (*p* < 0.001; Table 1 and Figure 1D). Participants with higher cumulative parasite density experienced their peak parasite density later during their infection (*p* < 0.001; Table 1).

**Table 1:** Characteristics of the study population according to categories of cumulative parasite density.

	All participants	Categories of cumulative parasite density		
		Low	Medium	High
*N*	54	19	17	18
**Time point^a^** (*N*)				
-C_-1_	45	15	14	16
-C_+30_	45	17	14	14
-C_+115_	32	8	11	13
Cumulative parasite density^b^ median (IQR)	8.3 (7.0–9.8)	6.2 (4.6–7.4)	8.7 (8.2–9.2)	10.0 (9.8–10.7)
Peak parasite density^c^ median (IQR)	6.9 (2.0–31.6)	1.0 (0.3–2.4)	7.4 (5.1–19.1)	42.6 (22.7–75.4)
Day of peak parasite density median (IQR)	10.4 (7.0–12.3)	7.0 (7.0–9.3)	9.6 (7.0–11.5)	12.2 (10.6–13.5)

*Categories of cumulative parasite density are based on tertiles of cumulative parasite density. IQR, interquartile range. ^*a*^Citrate plasma samples for antibody detection were selected at three time points: 1 day pre-challenge (C_-*1*_), 21 or 35 days after challenge (median 30 days; C_+*30*_) and 64, 140, or 213 days after challenge (median 115 days; C_+*115*_). All available samples were analyzed. ^*b*^Cumulative parasite density is expressed as the log-transformed area under the curve for parasites/μl versus time in days until the day of treatment. One participant was not treated until day 36, however, this participant did not have a sample available at C_+*30*_ for antibody detection thus their total cumulative parasite density was used for categorization in this table. ^*c*^Peak parasite density is the maximum number of parasites/μl during the infection until the day of treatment*.

### Minimal Number of Antigens to Detect Infection

**FIGURE 2:**
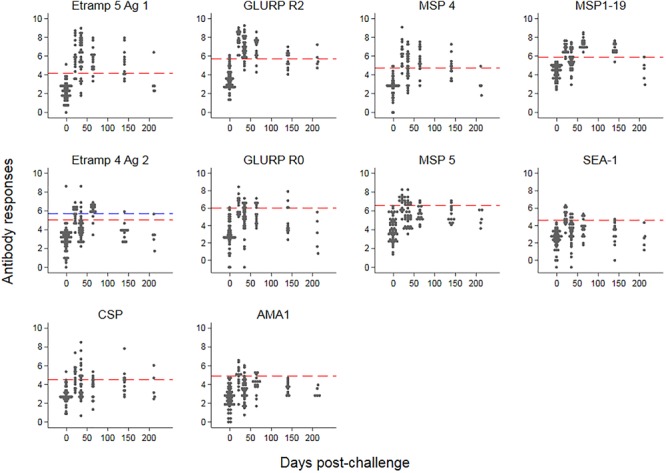
Kinetics of antimalarial antibody responses after controlled human malaria infection. Antibody responses (expressed as log-transformed median fluorescence intensity corrected for background reactivity) are shown over time, starting 1 day pre-challenge. Red dashed lines represent thresholds of seropositivity using the mean plus two standard deviations of pre-challenge responses across 45 participants. For Etramp 4 Ag 2, the outlier at baseline (blue triangle) was removed for the threshold calculation; the blue dashed line represents the threshold if the outlier at baseline was included.

**FIGURE 3:**
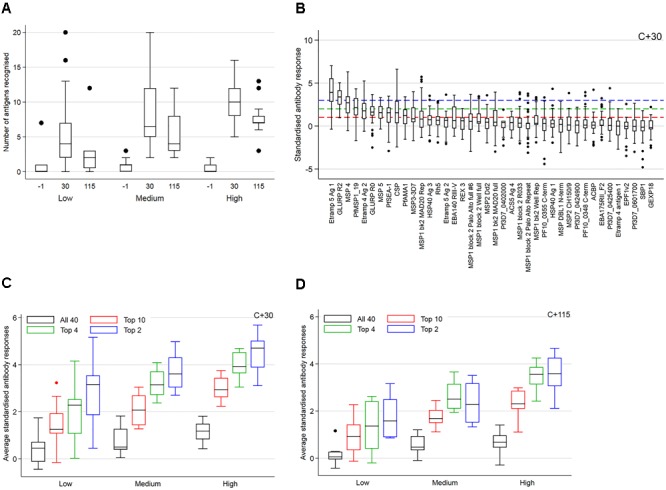
The number of antigenic targets recognized at three time points **(A)**, standardized antibody responses **(B)** and averaged standardized antibody responses **(C,D)**. In **(A,C,D)** results are shown over tertiles of cumulative parasite density (expressed as the log-transformed area under the curve for parasite density versus time until, and including the day of treatment). In **(A)** results are further categorized over time points post-challenge. An antigen is considered recognized if antibody responses (expressed as log-transformed median fluorescence intensity corrected for background reactivity) were higher than the mean plus two standard deviations (SD) of pre-challenge responses across 45 participants (i.e., seropositive). In **(B–D)** antibody responses were standardized by subtracting the mean of pre-challenge responses across 45 participants and dividing by its SD. In **(B)** standardized antibody responses 30 days post-challenge are ordered by median reactivity on the *x*-axis. Dashed lines represent arbitrary thresholds at 1 SD (red – top 10 antigenic targets), 2 SD (green – top 4 antigenic targets) or 3 SD (blue – top 2 antigenic targets) greater than the mean of pre-challenge responses. In **(C,D)** standardized antibody responses to antigens with a median above these arbitrary thresholds were averaged and are shown 30 days **(C)** and 115 days **(D)** after challenge, using the same color scheme. Average responses across all 40 targets are shown in black.

The kinetics of IgG responses following challenge are shown in Figure 2, while Figure 3A shows the number of antigenic targets recognized before and after challenge per category of cumulative parasite density. Two participants recognized more than five out of the panel of 40 antigens pre-challenge (C_-1_); 1 month post-challenge (C_+30_) one of these two participants recognized two additional antigens (9 antigens in total) while the other participant recognized thirteen additional antigens (20 antigens in total). For all categories of cumulative parasite density, the peak number of antigens recognized was at C_+30_. The proportion of targets recognized increased over categories of cumulative parasite density (*p* < 0.001 for C_+30_ and 2–7 months post-challenge; C_+115_). High seroprevalence (i.e., over 80%) was seen against GLURP-R2 (91%, 41/45) and Etramp 5 Ag 1 (82%, 37/45) at C_+30_, and against MSP1-19 at C_+115_ (84%, 27/32). For participants with medium to high cumulative parasite density, all were seropositive to GLURP-R2 and 96% to Etramp 5 Ag 1 (27/28) at C_+30_ (Supplementary Figure S2), and 96% responded to MSP1-19 at C_+115_ (23/24). For the lowest category, 77% (13/17) responded to GLURP-R2 and 59% (10/17) to Etramp 5 Ag 1 at C_+30_, and 50% to MSP1-19 at C_+115_ (4/8). Addition of one to two other antigenic targets for this low exposure category at either time point included all participants with an antibody response (see below for one non-responding participant).

### Strong Correlation Between Cumulative Parasite Density and Antibody Intensity

**FIGURE 4:**
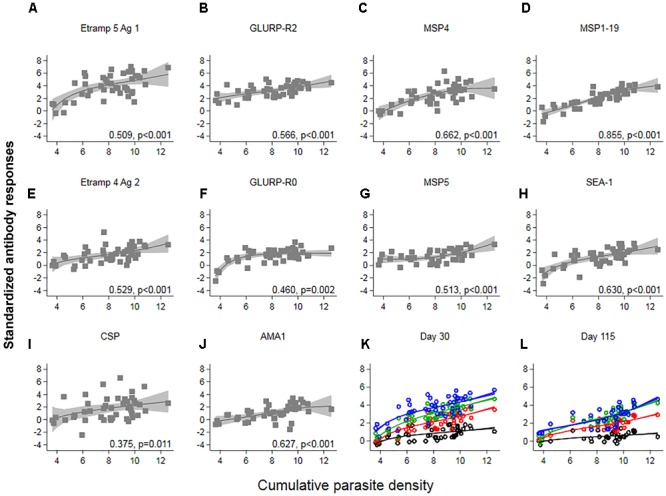
The association between cumulative parasite density and standardized antibody responses. Cumulative parasite density is expressed as the log-transformed area under the curve for parasite density versus time until, and including the day of treatment. Antibody responses (expressed as log-transformed MFI corrected for background reactivity) 30 days post-challenge were standardized by subtracting the mean of pre-challenge responses across 45 participants and dividing by its standard deviation (SD). In **(A–J)** Spearman’s ρ and associated *p*-values are shown per antigen. Solid lines and shaded areas are fractional polynomial fits with 95% confidence intervals. In **(K,L)** standardized antibody responses against antigenic targets with a median above arbitrary thresholds of 1 SD (red – top 10 antigenic targets), 2 SD (green – top 4 antigenic targets) or 3 SD (blue – top 2 antigenic targets) greater than the mean of pre-challenge responses, were averaged. Average responses across all forty targets are shown in black. In **(K)** Spearman’s ρ for all 40: 0.51, top 10: 0.77, top 4: 0.82, top 2: 0.61; *p* < 0.001 and in **(L)** all 40: 0.50, top 10: 0.69, top 4: 0.78, top 2: 0.72; *p* < 0.001.

Responses up to seven SD greater than the mean of C_-1_ responses were seen at C_+30_ for Etramp 5 Ag 1. Other targets associated with high antibody levels at C_+30_ were GLURP-R2, MSP4 and CSP (SD’s greater than five recorded). All participants showed a minimum of one SD greater than the mean of C_-1_ responses for GLURP-R2, while for all other antigens zero to negative responses were seen in at least one of the participants (Figure 3B). Highly reactive antigens at C_+30_ were those associated with median responses over arbitrary thresholds of three SD (Top 2: Etramp 5 Ag 1 and GLURP-R2), two SD (Top 4: top 2, as well as MSP4 and MSP1-19) or one SD (Top 10: top 4 as well as GLURP-R0, MSP5, SEA-1, CSP, Etramp 4 Ag 2, AMA1) greater than the mean of C_-1_ responses (Table 2). Standardized antibody responses to these top responding antigens were averaged to represent overall antibody density at C_+30_ (Figure 3C) and C_+115_ (Figure 3D). Most individual antigenic targets showed moderate correlation with parasite exposure (i.e., Spearman’s ρ = 0.50–0.69) except for GLURP-R0 and CSP (not significant at *p* > 0.00125), while MSP1-19 showed strong correlation (Spearman’s ρ = 0.86, *p* < 0.001), see (Figure 4). Overall antibody density of top responding antigens showed a strong correlation with cumulative parasite density at C_+30_ (all 40 antigens: Spearman’s ρ = 0.51, while top 4 responding antigens Spearman’s ρ = 0.82; *p* < 0.001) and C_+115_ (all 40 antigens: Spearman’s ρ = 0.50, while top 4 responding antigens Spearman’s ρ = 0.78; *p* < 0.001).

**Table 2:** Characteristics of the top 10 antigenic targets associated with the highest antibody responses 30 days post-challenge in controlled human malaria infection participants.

Gene ID	Description	Name	Allele	Location^a^	AA position	Expression tag	Reference
PF3D7_0532100	Early transcribed membrane protein 5	Etramp 5 Ag 1	3D7	iRBC/PVM	26–111	GST	Spielmann et al., 2003; Tetteh, unpublished
PF3D7_1035300	Glutamate rich protein R2	GLURP-R2	F32	Merozoite (Peripheral)	705–1178	His_x6_	Theisen et al., 1995
PF3D7_0207000	Merozoite surface protein 4	MSP4	D10	Merozoite surface (GPI-anchored)	43–107	GST	Marshall et al., 1997
PF3D7_0930300	19 kDa fragment of the merozoite surface protein-1	MSP1-19	Wellcome	Merozoite surface (GPI-anchored)	1631–1726	GST	Burghaus and Holder, 1994
PF3D7_0423700	Early transcribed membrane protein 4	Etramp 4 Ag 2	3D7	iRBC/PVM	76–136	GST	Spielmann et al., 2003; Tetteh, unpublished
PF3D7_1035300	Glutamate rich protein R0	GLURP-R0	F32	Merozoite (Peripheral)	94–489	His_x6_	Theisen et al., 1995
PF3D7_0206900	Merozoite surface protein 5	MSP5	3D7	Merozoite surface (GPI-anchored)	147–207	GST	Marshall et al., 1998
PF3D7_1021800	Schizont egress antigen 1	SEA-1	3D7	Schizont/Maurer’s cleft	810–1083	GST	Raj et al., 2014; Tetteh, unpublished
PF3D7_0304600	Circumsporozoite protein	CSP	3D7	Sporozoite surface	20–373	n/a	Kastenmüller et al., 2013
PF3D7_1133400	Apical membrane antigen 1	AMA1	FVO	Sporozoite/Merozoite (Micronemes)	97–546	His_x6_	Collins et al., 2007

*GPI, glycosylphosphatidylinositol; PVM, parasitophorous vacuolar membrane; iRBC, infected red blood cell; GST, glutathione S-transferase; kDa, kilodalton; AA, amino acid*. *^*a*^Location information is based on published literature and mass spec proteomic data (plasmodb)*.

### Participants With Limited Antibody Response

One participant had no demonstrable IgG to any of the 40 malarial antigens tested in samples at either of the post-challenge time points, though tetanus toxoid responses were recorded (log-transformed MFI-bkg values over 7.9 across both time points). For participants with serum samples available at both post-challenge time points, two other participants had an antibody response to only one of the forty antigens at either the C_+30_ or the C_+115_ time point (to CSP or GLURP-R2). The total proportion of individuals with no detectable IgG antibodies at C_+30_ was therefore 4.4% (2/45) and at C_+115_ 6.5% (2/31). These three participants were in the lowest category of cumulative parasite density and had peak parasite densities ≤0.20 parasites/μl.

## Discussion

Controlled human malaria infection trials provide unique opportunities to study immune responses after exposure to a known number of malaria-infected mosquitoes and an accurately quantified parasite exposure (Sauerwein et al., 2011; Scholzen and Sauerwein, 2016). We measured antimalarial antibody (IgG) responses in previously malaria naïve individuals from eight CHMI studies using a custom-made protein microarray. We showed that low parasite densities generated detectable IgG responses in 96% of the participants 1 month after challenge, and in 94% two to 7 months after challenge. Even at the low parasite densities recorded in these participants, a strong correlation was seen between cumulative parasite density and the number of antigenic targets recognized as well as the intensity of IgG responses. Immune responses to a subset of proteins including one hypothesized to be associated with recent exposure were developed by nearly all individuals (i.e., GLURP-R2, MSP1-19 and Etramp 5 Ag 1). It is an important observation that exposure to these infections was detected considering the low parasite density range, which would probably have remained undetected by routine microscopy or rapid diagnostic tests (i.e., 50/54 participants had peak parasite densities <100 parasites/μl while all remained <200 parasites/μl) (Wongsrichanalai et al., 2007; Okell et al., 2009; Wu et al., 2015).

We assessed IgG reactivity to forty purified recombinant antigens of the *P. falciparum* parasite. All targets were associated with the erythrocytic stage of the parasite life cycle, except for one pre-erythrocytic target (CSP). GLURP-R2, Etramp 5 Ag 1, MSP4 and MSP1-19 were associated with the highest relative antibody responses. Seroprevalence 1 month post-challenge was highest for GLURP-R2 (91%) and Etramp 5 Ag 1 (82%), dropping to approximately two-thirds seropositive two to 7 months post-challenge. Etramp 5 Ag 1 was associated with the highest antibody levels 1 month after challenge relative to pre-challenge responses. This antigen was one of the targets highlighted as a potential marker of recent exposure in a cohort of Ugandan children (Helb et al., 2015). Likewise, GLURP-R2 was associated with recent exposure in a Cambodian population (Kerkhof et al., 2016). Although we were unable to assess the rate of antibody decay in this study due to the small sample size, the low number of individuals with repeated samples and limited follow up time, high reactivity is evident in this previously non-exposed population. Other hypothesized markers of recent exposure identified by Helb et al. (2015) were also recognized in this population (42% for CSP and 27% for Etramp 4 Ag 2 1 month post-challenge), whereas HSP40 and GEXP18 were not (<5%). At 2–7 months after challenge, MSP1-19 was associated with the highest seroprevalence (84%; 27/32). This target also showed the strongest correlation with cumulative parasite density 1 month post-challenge (Spearman’s ρ = 0.86, *p* < 0.001). MSP1-19 has been associated with changes in transmission over time (van den Hoogen et al., 2015) and in recent transmission (Supargiyono et al., 2013). MSP4 was the final target in the top four responding antigens 1 month after challenge, while MSP5 was in the top 10. These antigens were associated with protection against clinical disease in Senegalese (Perraut et al., 2017), Brazilian (Medeiros et al., 2013), and Vietnamese (Wang et al., 2001) populations and warrant further investigation. MSP4 was also strongly associated with recent asymptomatic and symptomatic infections in Kenyan children alongside AMA-1, MSP1-19, and EBA140 RII (McCallum et al., 2017).

Previous studies describing antibody responses to malaria in CHMI participants concluded that a single CHMI is sufficient to induce production of antibodies directed against sporozoite, liver-stage and cross-stage antigens (Scholzen and Sauerwein, 2016). In line with our current findings, the magnitude of antibody and memory B-cell responses to cross-stage antigen MSP1-19 was reported to correlate with the degree of parasite exposure (duration and peak density) (Biswas et al., 2014; Elias et al., 2014; Nahrendorf et al., 2014; Walker et al., 2015). The majority of studies examining antibody responses following CHMI focused on antibody responses related to (sterile) protection and identified antigenic targets such as EXP-1 (Obiero et al., 2015), LSA-1 (Felgner et al., 2013; Nahrendorf et al., 2014), TRAP (Peng et al., 2016), and CSP (Felgner et al., 2013; Nahrendorf et al., 2014; Obiero et al., 2015; Hickey et al., 2016), showing some evidence for an increase in responses with increasing parasite exposure. Only a limited number of studies have reported antibody responses to multiple malarial antigenic targets, other than CSP or AMA1 and MSP1-19 alone, in the previously malaria naïve control groups of CHMI trials (Turner et al., 2011; Obiero et al., 2015; Hodgson et al., 2016). Obiero et al. (2015) and Hodgson et al. (2016) assessed antibody responses after CHMI in both non-endemic and endemic populations using ELISA (Hodgson et al., 2016). Seropositivity against MSP1-19, Rh5, CSP, and LSA-1 were similar to those we recorded. However, seropositivity against AMA-1 was considerably higher (Hodgson et al., 2016) or non-existent (Obiero et al., 2015), compared to moderate responses in our study. These differences may be due to differences in assay protocols, strains used [3D7 (Hodgson et al., 2016) versus FVO] or the levels of parasite exposure. In both studies, antimalarial antibody responses were induced more efficiently in endemic volunteers compared to non-endemic volunteers, even if baseline responses were the same, indicating the presence of memory B cells in endemic populations (Wipasa et al., 2010). Burel et al. (2017) reported that the generation of antibodies to blood-stage antigens following CHMI is strongly influenced by expression patterns of microRNA, which are involved in regulation of immune responses, and that microRNA expression patterns vary considerably between individuals. Turner et al. (2011) explored antibody responses to (multiple regions of) five blood-stage antigenic targets in volunteers from a Dutch CHMI trial using a multiplex bead assay. They found responses in 93% of volunteers 35 days post-challenge, mostly against PfEMP1 (which was not included in our panel) and GLURP-R2, with lower seropositivity against GLURP-R0 and MSP3, similar to our results. They saw no association between PfEMP1 antibody acquisition and parasite load or maximum parasite density (Turner et al., 2011). The strong correlations seen in the current cohort are most likely due to the increased range in parasite exposure. In addition to the overlap in these previously described results, we show responses to a range of erythrocytic antigens including those potentially associated with recent infection using a newly developed custom-made protein microarray.

Studying antibody responses following malaria infections in volunteers from non-endemic areas is informative due to difficulties in assessing the exact level and frequency of previous exposure in endemic populations. Genetic and environmental differences between malaria endemic and non-endemic populations make direct translation of these results challenging. Moreover, parasite densities are likely to reach considerably higher levels in natural infections, which in turn would influence the number of antigenic targets recognized and the intensity of existing responses. Nevertheless, an overlap was seen between our results and those from endemic populations for certain antigenic targets [Etramp 5 (Helb et al., 2015), Etramp 4 (Helb et al., 2015), CSP (Baum et al., 2013; Helb et al., 2015), MSP4 (Baum et al., 2013; Burel et al., 2017; McCallum et al., 2017), MSP1-19 (McCallum et al., 2017) and to a lesser extent in our results AMA-1 (Stanisic et al., 2015; McCallum et al., 2017)], while not for other targets [GEXP18 (Helb et al., 2015), HSP40 (Helb et al., 2015), EBA140, EBA175, and MSP2 (Stanisic et al., 2015; Burel et al., 2017; McCallum et al., 2017]). Overall, it is an important observation that antibody responses could be detected in a previously non-exposed population following such low-density infections. The duration of detectable antibody titres in the current study population, especially after re-infection, is unknown. This information is of use for validation of these targets in sero-surveillance aiming to generate proxy estimates of incidence for transmission monitoring. Further assessment of antibody kinetics following infection (i.e., assessing time since infection) using these antigenic targets is essential. Although all participants were previously naïve for malaria, we saw reactivity to some of the included targets at the pre-challenge time point (i.e., Etramp 4 Ag 2) potentially due to cross-reactivity with antigens from other pathogens. Likewise, responses to AMA1 and MSP1-19 were seen in some of the malaria naïve United Kingdom adults pre-challenge at similar concentrations to those detected at C_+30_ (Hodgson et al., 2016). Using a two-standard deviation rule to define the threshold of positivity has its limitations; by default, this will cause some participants to be defined as seropositive pre-challenge (i.e., approximately 2.5%). One participant remained undetected for IgG responses against the panel of 40 antigens evaluated. This may be due to the very low exposure to malaria parasites as their peak parasite density was the lowest recorded (0.15 parasites/μl) and the duration of their infection as detected by qPCR was 1 day. Two other participants with limited antibody responses recorded (i.e., to one of the antigens in the panel at only one of the time points) had peak parasite densities of ≤0.20 parasites/μl. Furthermore, the panel of targets evaluated is finite and there may be antigens not yet expressed that would have induced a detectable immune response in these participants. Lastly, IgM responses were not evaluated in this study, which may also have been present in these participants.

## Conclusion

Antibody responses to erythrocytic antigens were detectable following low-density experimental *P. falciparum* infections in nearly all volunteers. This included antigenic targets potentially related to recent infection (Helb et al., 2015) as well as well-known targets such as AMA1, MSP1-19, and CSP. Detecting exposure to recent infections below the detection limit of conventional diagnostics is essential to interrupt transmission (Ouédraogo et al., 2016; malERA Refresh Consultative Panel on Characterising the Reservoir and Measuring Transmission, 2017), especially in low transmission and elimination settings where the majority of infections are of low-density (Okell et al., 2009; Wu et al., 2015). We showed a strong dose-response relationship between cumulative parasite density and antibody density across multiple targets. Moving forward, a selection of 4–5 targets could be combined in a field-based assay such as an ELISA to rapidly assess remaining transmission in (near-) eliminating settings. This would be advantageous compared to measuring infection rates, as PCR-based techniques are more costly and labor-intensive, require larger sample sizes in low transmission settings, are more sensitive to fluctuations in parasite densities during an infection and overall rates between seasons. Ultimately, our findings require validation in endemic populations to define the minimum set of antigens needed to reliably detect exposure to natural infections.

## Author Contributions

LH, JW, TO, IR, TB, CD, RS, and KT designed the study. JW, IR, TB, and RS were involved in the design and performance of the original CHMI studies, and collection of all samples. LR, JB, RC, SS, SD, and KT provided antigen constructs. TO and KT designed the assay. LH and TO processed samples. LH, JW, IR, TB, CD, and KT performed data analyses and interpreted results. LH drafted the manuscript with support from JW, TO, IR, JB, TB, CD, RS, and KT. All authors read and approved the final manuscript.

## Conflict of Interest Statement

The authors declare that the research was conducted in the absence of any commercial or financial relationships that could be construed as a potential conflict of interest.
