# Supplementary material for: Antibody Responses to Antigenic Targets of Recent Exposure Are Associated With Low-Density Parasitemia in Controlled Human Plasmodium falciparum Infections
Source: Front Microbiol. 2019 Jan 16;9:3300. doi: 10.3389/fmicb.2018.03300 (PMC6343524; doi:10.3389/fmicb.2018.03300)
Supplement: Supplementary file 1 [file Table_1.DOCX]

**Supplementary Table 1: Controlled human malaria infection trials included in the study.** Samples from participants across eight controlled human malaria infection trials were analysed in the current study.

| **Study** | **Year** | **NCT number** | **Ethical approval CCMO** | **Reference** |
| --- | --- | --- | --- | --- |
| 1 | 2011 | NCT01236612 | NL34273.091.10 | [24] |
| 2 | 2011 | NCT01218893 | NL33904.091.10 | [25] |
| 3 | 2012 | NCT01422954 | NL 37563.058.11 | [26] |
| 4 | 2012 | NCT01728701 | NL39541.091.12 | [9] |
| 5 | 2015 | NCT02080026 | NL48301.091.14 | - |
| 6 | 2015 | NCT02098590 | NL48732.091.14 | [23] |
| 7 | 2015 | NCT02098590 | NL48732.091.14 | [23] |
| 8 | 2016 | NCT02836002 | NL56659.091.16 | [27] |

NCT: National Clinical Trial. CCMO: Central Committee on Research Involving Human Subjects.
