# Supplementary material for: Antibody Responses to Antigenic Targets of Recent Exposure Are Associated With Low-Density Parasitemia in Controlled Human Plasmodium falciparum Infections
Source: Front Microbiol. 2019 Jan 16;9:3300. doi: 10.3389/fmicb.2018.03300 (PMC6343524; doi:10.3389/fmicb.2018.03300)
Supplement: Supplementary file 3 [file Data_Sheet_1.docx]

**Supplementary Figure 1: Duplicate median fluorescence intensity measures from repeated spots on the protein micro-array.** Paired measurements across all the samples tested are shown; duplicate measures were averaged for analyses. The grey line represents the line of equality. Pearson’s ρ=0.99, p<0.001.

**Supplementary Figure 2: Antibody responses over categories of cumulative parasite density thirty days post-challenge.** Cumulative parasite density is expressed as the log-transformed area under the curve for parasite density versus time until, and including the day of treatment. Tertiles were used to categorise low, medium and high. Antibody responses are expressed as log-transformed median fluorescence intensity corrected for background reactivity. Red dashed lines represent thresholds of seropositivity using the mean plus two standard deviations of pre-challenge responses across 45 participants. For Etramp 4 Ag 2, the outlier at baseline was removed for the threshold calculation; the blue dashed line represents the threshold if the outlier at baseline was included.
